# Metagenome-Sourced Microbial Chitinases as Potential Insecticide Proteins

**DOI:** 10.3389/fmicb.2019.01358

**Published:** 2019-06-18

**Authors:** Francesca Berini, Morena Casartelli, Aurora Montali, Marcella Reguzzoni, Gianluca Tettamanti, Flavia Marinelli

**Affiliations:** ^1^Laboratory of Microbial Biotechnology, Department of Biotechnology and Life Sciences, University of Insubria, Varese, Italy; ^2^Laboratory of Insect Physiology and Biotechnology, Department of Biosciences, University of Milan Milan, Italy; ^3^Laboratory of Invertebrate Biology, Department of Biotechnology and Life Sciences, University of Insubria Varese, Italy; ^4^Laboratory of Human Morphology, Department of Medicine and Surgery, University of Insubria Varese, Italy

**Keywords:** insecticidal proteins, chitinase, metagenomics, heterologous expression, *Streptomyces*, insect control, *Bombyx mori*, peritrophic matrix

## Abstract

Microbial chitinases are gaining interest as promising candidates for controlling plant pests. These enzymes can be used directly as biocontrol agents as well as in combination with chemical pesticides or other biopesticides, reducing their environmental impact and/or enhancing their efficacy. Chitinolytic enzymes can target two different structures in insects: the cuticle and the peritrophic matrix (PM). PM, formed by chitin fibrils connected to glycoproteins and proteoglycans, represents a physical barrier that plays an essential role in midgut physiology and insect digestion, and protects the absorptive midgut epithelium from food abrasion or pathogen infections. In this paper, we investigate how two recently discovered metagenome-sourced chitinases (Chi18H8 and 53D1) affect, *in vitro* and *in vivo*, the PM integrity of *Bombyx mori*, a model system among Lepidoptera. The two chitinases were produced in *Escherichia coli* or, alternatively, in the unconventional – but more environmentally acceptable – *Streptomyces coelicolor*. Although both the proteins dramatically altered the structure of *B. mori* PM *in vitro*, when administered orally only 53D1 caused adverse and marked effects on larval growth and development, inducing mortality and reducing pupal weight. These *in vivo* results demonstrate that 53D1 is a promising candidate as insecticide protein.

## Introduction

Pesticides derived from chemical synthesis are massively used to control different pests that constantly threaten crop production (Atwood and Paisley-Jones, 2017). The main drawbacks of chemically synthesized pesticides are their broad toxicity and accumulation into ecosystems and food chains (Kumar et al., 2019). Alternatively, biocontrol or biological control, i.e., the use of organisms or their products (biopesticides), is favored by the better selectivity of these agents toward the target pests, their biodegradability, and reduced toxicity (Czaja et al., 2015; Bonanomi et al., 2018; Damalas and Koutroubas, 2018). In contrast, the successful use of biocontrol agents is often limited by their instability and scarce persistence into environment, as well as by their slower mode of action and reduced efficacy in comparison to chemical pesticides. Bacteria and fungi exhibiting fungicidal, insecticidal, and/or nematicidal action are commonly used as biocontrol agents. They produce antibiotics and secrete a variety of hydrolytic enzymes (chitinases, proteases, lipases, and glucanases), which concur in disrupting essential structures for pathogen life. A compelling alternative is formulating cocktails of (semi)purified antibiotics and enzymes, which mimic living biocontrol agents, without presenting the limitations inherent to their use and storage. Such biopesticides can be used alone or in combination with other controlling agents to enhance their efficacy (Regev et al., 1996; Karasuda et al., 2003; Liu et al., 2010). If added to chemically synthesized pesticides, biopesticides might allow the reduction of their dosage, alleviating their negative impact on the ecosystem (Karasuda et al., 2003). To this purpose, chitinases represent promising biopesticides, since they hydrolyze chitin, which is present in different plant pests, i.e., insects, fungi, and nematodes (Mavromatis et al., 2003; Neeraja et al., 2010; Hjort et al., 2014; Soares et al., 2015; Berini et al., 2016, 2017b, 2018). Additionally, they are harmless for plants and vertebrates, which do not possess chitin in their tissues. Chitin is a linear homopolymer of *N*-acetylglucosamine (GlcNAc) and exerts fundamental roles in the vital structures of pests. It is a structural component of cell wall in fungi, of eggshell in nematodes, and of both cuticle and peritrophic matrix (PM) in insects. PM is a thin acellular sheath formed by chitin, glycoproteins, and proteoglycans, which lines the midgut epithelium of most insects (Hegedus et al., 2009; Berini et al., 2018). Chitinases belong to the family of glycosyl hydrolases. Based on their mode of action on chitin, they are classified as endochitinases, which split chitin randomly at internal sites, or as exochitinases that remove monomers (β-*N*-acetyl glucosaminidases) or dimers (chitobiosidases) of GlcNAc from the non-reducing end of chitin chains (Adrangi and Faramarzi, 2013; Berini et al., 2018).

In the recent years, we applied function- and/or sequence-based screening approaches to different metagenomes for discovering novel bioactive chitinases of microbial source, which differ from those already known that have been discovered by classical microbiological methods (Hjort et al., 2014; Cretoiu et al., 2015; Berini et al., 2017b). Since the vast majority of microorganisms present in natural samples (up to 99–99.9%) are recalcitrant to cultivation, metagenomics, being culture-independent, facilitates the task of encrypting novel chitinases (Berini et al., 2017a). Thanks to this approach, two of the first metagenomics-sourced chitinases were recently discovered: Chi18H8 was identified in 2014 from a naturally phytopathogen-suppressive soil in Sweden (Hjort et al., 2014; Berini et al., 2017b), whereas 53D1 was identified in 2015 in a chitin-supplemented agricultural soil from an experimental farm in the Netherlands (Cretoiu et al., 2015). Few milligrams of both chitinases were initially produced in *Escherichia coli* as heterologous host and partially biochemically/functionally characterized. Interestingly, Chi18H8 showed antifungal activity toward the phytopathogen fungi *Fusarium graminearum* and *Rhizoctonia solani* (Hjort et al., 2014; Berini et al., 2017b), whereas 53D1 looked interesting since it was markedly stable in a wide range of conditions, including in the presence of high salt concentrations (Cretoiu et al., 2015). We recently described the development to a 30-L bioreactor pilot scale of an effective process to produce Chi18H8 by mild solubilization of inclusion bodies (IBs) in *E. coli* (Berini et al., 2017b). Herein, we describe the optimization of 53D1 production by using an alternative heterologous host – the Gram-positive bacterium *Streptomyces coelicolor* A3(2) – and report on producing and testing both Chi18H8 and 53D1 as insecticidal proteins in *Bombyx mori*, a reference model among Lepidoptera. To our knowledge, this is the first investigation on the insecticidal activity of metagenome-sourced chitinases, which might represent promising candidates as biocontrol agents.

## Materials and Methods

### *53D1* Gene Cloning

The nucleotide sequence of the metagenomic fosmid insert that includes *53D1* chitinase gene was deposited in the GenBank database (accession number LN824156.1) (Cretoiu et al., 2015). The chitinase-encoding cDNA was sub-cloned into the multi-copy expression vector pIJ86 (Binda et al., 2013) (kindly gifted by M. J. Bibb, John Innes Centre, Norwich, United Kingdom) under the control of the constitutive *ermE^∗^* promoter, by using the fosmid DNA as template. Primers used for amplification were pIJ86_53D1_FW (5′ ATATGGATCCGTATGAAGGAGGTCATTCATGAGTCACGGTTCGGTC 3′) and pIJ86_53D1_RV (5′ ATTAAAGCTTCTAGTGGTGGTGGTGGTGGTGCGGTCTCAGCCGGGA 3′), including the restriction sites (underlined) for *Bam*HI and *Hin*dIII, respectively, and introducing a C-terminal His_6_-Tag in the recombinant protein. All cloning procedures were carried out in *E. coli* DH5α (Invitrogen-Life Technology, Carlsbad, CA, United States). The construct was checked by DNA sequencing (BMR Genomics, Padua, Italy) and transformed into the non-methylating *E. coli* ET12567/pUZ8002 cells (Marcone et al., 2010b). Luria-Bertani (LB, Sigma-Aldrich, St. Louis, MO, United States) agar plates were used for propagating *E. coli* strains.

Intergeneric conjugation between the *E. coli* donor and the recipients *S. coelicolor* A3(2), *S. venezuelae* ATCC 10595, and *S. lividans* TK24 was conducted following the protocol reported in Binda et al. (2013). Transformation of the recombinant *Streptomyces* spp. was checked by colony PCR (Binda et al., 2013). pIJ86_53D1_FW and pIJ86_53D1_RV primers were used to verify ex-conjugants carrying pIJ86::*53D1* plasmid. Primers pIJ86_FW (5′ TGCACGCGGTCGATCTTGAC 3′) and pIJ86_RV (5′ TCATGGTCGGTCTCCTGGTG 3′), annealing to regions of the vector around the multiple cloning site, were used to check transformation with the empty pIJ86 vector.

### 53D1 Heterologous Production

Reagents were purchased from Sigma-Aldrich, St. Louis, MO, United States, unless otherwise indicated. Mannitol soya flour (MS) agar medium (Kieser et al., 2000) was used for propagating *Streptomyces* spp. Streptomycetes were stored for long-term preservation as spores in 10% (v/v) glycerol. For cultivating the recombinant strains, agar plates and liquid media were always supplemented with 50 μg/mL apramycin. Strains were reactivated by growing them for 72 h into 100-mL Erlenmeyer flasks containing 20 mL AurM medium (in g/L: 20 maltose, 10 dextrin, 15 soybean meal, 4 casein enzymatic hydrolysate, 4 bacteriological peptone, 2 yeast extract, 2 CaCO_3_, pH 7.0) (Marcone et al., 2010b). Three hundred milliliters baffled flasks containing 50 mL YEME (yeast extract – malt extract, in g/L: 3 yeast extract, 5 bacteriological peptone, 3 malt extract, 20 glucose, pH 7.0) (Binda et al., 2013) were then inoculated at 10% (v/v) and further shaken at 200 revolutions per minute (rpm) at 28°C for 72 h. Finally, 500-mL baffled flasks containing 100 mL of five different production media (commonly used for streptomycetes) were inoculated at 10% (v/v), incubated at 200 rpm and 28°C for 240 h. Liquid production media used were YEME, MV (medium V) (Marcone et al., 2010a), R5 medium (Kieser et al., 2000), TSB (tryptone soya broth) (Kieser et al., 2000), and Bennett’s medium (Dalmastri et al., 2016). All media were supplemented with 20 g/L glucose, if not already included, in order to repress the endogenous chitinolytic system of streptomycetes (Berini et al., 2018).

Every 24 h, 10 mL of culture broth were centrifuged at 1900 × *g* for 10 min at 4°C. Cell-free culture broths were collected and pH and residual glucose were measured by pH Test Strips 4.5–10.0 and Diastix strips (Bayer, Leverkusen, Germany). Secreted 53D1 production was estimated in cell-free culture broths by western blot analysis [after protein concentration by 10% (v/v) trichloracetic acid precipitation] and fluorimetric enzyme activity assay (see below). In parallel, cell pellets were recovered and biomass production was measured as wet weight. Then, pellets were sonicated on ice with 10–15 cycles of 30 s each (interposed with 30-s intervals), using a Branson Sonifier 250 (Dansbury, CT, United States) in 20 mM sodium acetate pH 5.0 supplemented with 10 μg/mL deoxyribonuclease (DNase) and 0.19 mg/mL phenylmethylsulfonylfluoride (PMSF). To remove insoluble material, centrifugation at 20,000 × *g* for 40 min at 4°C followed. Production of intracellular 53D1 was checked in the soluble fractions by western blot analysis and fluorimetric enzyme activity assay (see below).

### 53D1 Purification

For 53D1 purification, *S. coelicolor*/pIJ86::*53D1* was grown for 192–240 h in YEME medium. Proteins secreted in the cell-free culture broth were precipitated by slowly adding 80% (w/v) ammonium sulfate. After 2 h incubation at 4°C, centrifugation at 12,000 × *g* at 4°C for 40 min followed. The pellet was re-suspended in 1/5 (v/v) of 20 mM Tris–HCl pH 8.0 and dialyzed against the same buffer. The recombinant protein was purified onto a 5-mL Ni^2+^-Hitrap chelating affinity column (1.6 cm × 2.5 cm; GE Healthcare Sciences, Little Chalfont, United Kingdom), according to manufacturer’s instructions. The column was equilibrated with 20 mM Tris–HCl pH 8.0, 500 mM NaCl, and 20 mM imidazole. After extensive washing, the recombinant protein was eluted with 20 mM Tris–HCl pH 8.0, 500 mM NaCl, and 250 mM imidazole, followed by dialysis for 3 h against 20 mM sodium acetate pH 5.0. The purified protein was finally concentrated with 30 K Amicon Ultra-2 centrifugal filter devices (Merck KGaA, Darmstadt, Germany).

### Chi18H8 Production and Purification

Chi18H8 production in *E. coli* BL21 Star^TM^ (DE3), carrying the pET24b(+)::*chi18H8* expression plasmid, and its solubilization from IBs were accomplished as previously described (Berini et al., 2017b). In brief, to prepare the protein used in this work, *E. coli* BL21 Star^TM^ (DE3)/pET24b(+)::*chi18H8* cells were grown in 300-mL baffled Erlenmeyer flasks containing 80 mL LB medium supplemented with 50 μg/mL kanamycin, incubated overnight at 37°C and 200 rpm. Two liters flasks with 750 mL selective LB medium were inoculated with the pre-cultures (initial OD_600 nm_ = 0.1), and incubated at 37°C and 200 rpm until OD_600 nm_ reached 0.6. Protein production was induced by adding 0.4 mM isopropyl β-D-1-thiogalactopyranoside (IPTG) and cultivation was prolonged at 20°C for further 24 h.

Cells were harvested by centrifugation and re-suspended in 50 mM Tris–HCl pH 8.0, 25% (w/v) sucrose, and 1 mM ethylenediaminetetraacetic acid (EDTA). After incubation for 30 min at room temperature and vigorous shaking, samples were sonicated (six cycles of 30 s each). A total of 0.2 M NaCl, 1% (w/v) sodium deoxycholate (DOC), and 1% (v/v) Nonidet P-40 were added; the samples were further incubated as above and centrifuged (20,000 × *g* at 4°C for 30 min). The pellet was washed with 1% (v/v) Triton X-100 and 1 mM EDTA and centrifuged (12,000 × *g* at 4°C for 10 min). IB washing with this buffer was repeated twice, followed by washing with deionized water. After overnight storage at −20°C, the frozen pellet was resuspended in 10 mM lactic acid (10 mL/g cell) and incubated at 37°C and 200 rpm for 5 h. Centrifugation at 1900 × *g* at 4°C for 5 min was employed for removing insoluble material. Finally, solubilized Chi18H8 was dialyzed overnight against 20 mM sodium acetate pH 5.0.

### SDS-PAGE Electrophoresis and Western Blot

Protein fractions were analyzed by sodium dodecyl sulfate polyacrylamide (12% w/v) gel electrophoresis (SDS-PAGE), using a Tris-glycine system and Coomassie brilliant blue R-250 straining. For western blot analysis, anti His-Tag Antibody HRP conjugate (Novagen Inc., Madison, WI, United States) and chemiluminescence (ECL Western Blotting Detection System, GE Healthcare Sciences, Little Chalfont, United Kingdom) were used for protein identification.

### Chitinase Activity Assays

Chitinase activities were assayed by using the fluorimetric chitooligosaccharide analogs 4-methylumbelliferyl *N*-acetyl-β-D-glucosaminide (4-MU-GlcNAc), 4-methylumbelliferyl *N*,*N*′-diacetyl-β-D-chitobioside [4-MU-(GlcNAc)_2_], and 4-methylumbelliferyl *N,N*′*,N*″-triacetyl-β-D-chitotrioside [4-MU-(GlcNAc)_3_] (Cretoiu et al., 2015). Activity on these synthetic compounds was assayed in 100 mM sodium acetate pH 5.0, at 37°C. Chitinolytic activity was also determined on colloidal chitin as described in Berini et al. (2016). In this case, activity was measured at pH 3.0, 5.0, 7.0, or 9.0, by adjusting colloidal chitin’s pH with 0.1 M NaOH. One unit (U) of chitinase activity was defined as the amount of enzyme required for the release of 1 μmol of 4-MU or of GlcNAc per min at 37°C. The control of protease or lipase activities in purified 53D1 and Chi18H8 preparations was conducted as described in Berini et al. (2016).

### Experimental Insects

Larvae of *B. mori* [polyhybrid strain (126 × 57) (70 × 90)] were provided by CREA – Honeybee and Silkworm Research Unit (Padua, Italy). Insects were reared on artificial diet (Cappellozza et al., 2005) at 25 ± 0.5°C, under a 12:12 light-dark photoperiod, with 70 ± 5% relative humidity. Once insects had reached the last larval instar, they were staged and synchronized (see Franzetti et al., 2012 for details).

### Ultrastructural Analysis of the PM

#### Isolation of the PM and *in vitro* Incubation With Chi18H8 or 53D1 Chitinases

On second day of the fifth instar, larvae were anaesthetized with CO_2_. Midgut was isolated by cutting the insect dorsally and the PM was carefully separated from the midgut epithelium. The lumen content was removed from the PM by rinsing the matrix with PBS (phosphate-buffered saline, 137 mM NaCl, 2.7 mM KCl, 4.3 mM Na_2_HPO_4_, 1.4 mM KH_2_PO_4_, pH 7.0). Each sample was divided into four pieces and transferred into a 24-multiwell plate: two pieces were treated with Chi18H8 or 53D1 (40.5 U_tot_ per well, calculated as the sum of chitobiosidase and endochitinase activities on 4-MU-(GlcNAc)_2_ and 4-MU-(GlcNAc)_3_, respectively, in 100 mM sodium acetate buffer pH 5.0, while the other two were incubated in the same buffer in the absence of chitinases (controls). All the samples were processed for electron microscopy analysis.

#### Scanning Electron Microscopy (SEM)

After incubation with 53D1 or Chi18H8, PM was fixed with 4% (v/v) glutaraldehyde in 0.1 M sodium cacodylate buffer pH 7.4, overnight at 4°C. After post-fixation with 1% (w/v) osmium tetroxide and 1.25% (w/v) potassium ferrocyanide for 1 h, samples were dehydrated in an ethanol series and then incubated in hexamethyldisilazane (two steps of 10 min each). Samples were mounted on stubs, carbon coated with a Sputter K250 coater, and finally observed with a SEM-FEG XL-30 microscope (Philips, Eindhoven, Netherlands).

#### Transmission Electron Microscopy (TEM)

To analyze the samples at TEM, PM was fixed with 4% (v/v) glutaraldehyde in 0.1 M sodium cacodylate buffer pH 7.4, overnight at 4°C and then post-fixed with 1% (w/v) osmium tetroxide for 1 h. After dehydration in an ethanol series, specimens were embedded in an Epon/Araldite 812 mixture. Ultra-thin sections were obtained with Leica Reichert Ultracut S (Leica, Nußloch, Germany), then stained with lead citrate and uranyl acetate, and finally observed with a JEM-1010 transmission electron microscope (Jeol, Tokyo, Japan). Images were acquired with a Morada digital camera (Olympus, Münster, Germany).

### Bioassays With Chi18H8 and 53D1 Chitinases

After hatching, larvae were reared as reported in the Section “Experimental Insects,” and fed *ad libitum* with small pieces of artificial diet (1 cm × 1 cm × 1 mm), each overlaid with an equal volume (65 μL) of Chi18H8 or 53D1 (6 U_tot_/cm^2^ diet) dissolved in 100 mM sodium acetate, pH 5.0. Control larvae were grown on small pieces of artificial diet overlaid with the same volume of sodium acetate buffer. The diet was replaced every day. Different parameters were recorded: larval mortality (reported as percentage of the initial number of larvae), length of the larval stage (from hatching to the occurrence of wandering behavior), and weight of the pupae (evaluated on the eighth day of the pupal stage). For bioassays with 53D1, maximal larval weight before pupation and cocoon weight (measured on the eighth day of the pupal stage) were registered, too. Developmental stages of *B. mori* were defined according to Franzetti et al. (2012). Bioassays were performed in triplicate, by using at least 11 larvae for each experimental group. PM samples from larvae at the second day of the fifth instar reared on diet overlaid with 53D1 and relative controls were collected and processed for the analysis at SEM and TEM, as reported in the Section “Ultrastructural Analysis of the PM.”

### *In vitro* Incubation of Chitinases With Midgut Juice

Midgut juice was extracted from larvae at the second day of the fifth instar. Insects were anaesthetized with CO_2_, whole midguts were dissected and their luminal content was collected into a centrifuge tube. Centrifugation at 15,000 × *g* for 10 min was performed to remove insoluble material. Supernatants were aliquoted, stored at −80°C, and used within 2 weeks. Six U_tot_ of Chi18H8 or 53D1 were incubated at 25°C in 100 mM Tris–HCl pH 8.0 (control) or in the presence of different dilutions of the midgut juice (undiluted, or diluted 1:10 or 1:100 in 100 mM Tris–HCl pH 8.0). Aliquots were withdrawn at regular intervals up to 8 h and the residual chitobiosidase activity was measured using 4-MU-(GlcNAc)_2_ as substrate, according to the standard protocol described in the Section “Chitinase Activity Assays.”

## Results

### Production and Characterization of Chi18H8

**FIGURE 1:**
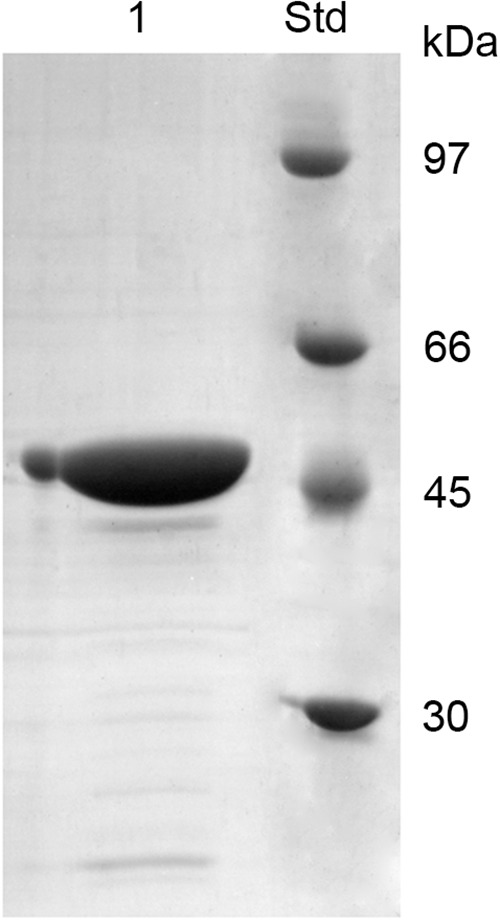
SDS-PAGE of Chi18H8 solubilization from *E. coli* BL21 Star^TM^ (DE3)/pET24b(+)::*chi18H8* IBs. 1, solubilized Chi18H8; Std, standard reference proteins.

Chi18H8 is a protein of 424 amino acids with a predicted molecular mass of 45.96 kDa and a theoretical isoelectric point of 7.75. To assay its insecticidal activity, Chi18H8 was produced in 2-L flasks and recovered from *E. coli* BL21 Star^TM^ (DE3)/pET24b(+)::*chi18H8* cells (Table 1) by using a newly developed process based on the mild solubilization of IBs, as recently described in Berini et al. (2017b). Following purification, Chi18H8 migrated in SDS-PAGE gels as a single band of ca. 47 kDa (46.77 kDa is the expected molecular mass for the recombinant His_6_-tagged protein). Protein purity was estimated to be >85% (Figure 1).

**Table 1:** Purification of Chi18H8 (A) from *E. coli* BL21 Star^TM^ (DE3)/pET24b(+)::*chi18H8* IBs and 53D1 (B) from *S. coelicolor*/pIJ86::*53D1* culture broth.

Purification step	Volume (mL)	Total proteins (mg)	Total activity (U)	Specific activity (U/mg protein)	Purification (-fold)	Yield (%)
*(A)*						
IBs	10.6	123.0	33.4	0.27	1.0	100.0
Soluble fraction from IBs	53.0	71.8	2623.0	36.7	78.5	84.0
*(B)*						
Crude broth	1000.0	1125.0	1080.0	0.96	1.0	100.0
Ammonium sulfate precipitation	200.0	1090.0	1067.1	0.98	1.0	98.8
Affinity chromatography	114.1	34.9	956.6	27.4	28.5	88.5

*For both proteins, data are relative to cells (Chi18H8) or cell-free culture broth (53D1) from 1 L of culture. Activity was assayed on 4-MU-(GlcNAc)_2_ as substrate, in 100 mM sodium acetate pH 5.0.*

Fluorimetric enzyme assay using standard synthetic oligosaccharides confirmed the Chi18H8 prevalent chitobiosidase activity on 4-MU-(GlcNAc)_2_ (37.92 ± 1.17 U/mg protein), its weaker endochitinase activity on 4-MU-(GlcNAc)_3_ (8.91 ± 1.72 U/mg protein), and none β-*N*-acetyl-glucosaminidase activity on 4-MU-GlcNAc. As reported in Table 2, pure Chi18H8 was able to hydrolyze colloidal chitin – a substrate that, although soluble, resembles the chemical structure of the naturally occurring insoluble chitin – with a maximum activity of about 1.47 ± 0.25 U/mg protein at pH 5.0. At pH 3.0, 7.0, and 9.0, ca. 22, 83, and 72% of the maximum activity were maintained, respectively (Table 2). None protease or lipase activity (lipases and proteases are enzymes usually secreted by streptomycetes that could interfere with the following insecticide assays) was detected in the enzyme preparation (data not shown).

**Table 2:** Chi18H8 and 53D1 activity on colloidal chitin at different pHs (mean ± standard error from at least three independent experiments).

pH	Chi18H8 (U/mg protein)	53D1 (U/mg protein)
3.0	0.32 ± 0.07	2.75 ± 0.35
5.0	1.47 ± 0.25	10.15 ± 1.40
7.0	1.22 ± 0.01	7.10 ± 0.20
9.0	1.06 ± 0.04	7.00 ± 0.10

### Heterologous Expression of 53D1 in *Streptomyces* spp.

*53D1* gene (63.03% G+C) consists of 1191 nucleotides coding for a protein of 396 amino acids with a predicted molecular mass of 43.60 kDa and a theoretical isoelectric point of 4.83. When cloned and expressed in *E. coli*, >80% of the recombinant protein accumulated as inactive form in insoluble fractions. Despite many efforts, we could not develop a protocol for solubilizing 53D1 in a biologically active form from IBs, as we did for Chi18H8. In addition, as reported in Cretoiu et al. (2015), the recovery yield of the soluble active form of 53D1 from *E. coli* cytoplasmic fraction was too low (no more than 0.60 mg/L culture and 0.12 mg/g cell) to support its further trials as insecticide protein. Thus, in this paper we report an alternative expression platform using soil Gram-positive actinomycetes belonging to the genus *Streptomyces* as heterologous hosts for 53D1 production.

**FIGURE 2:**
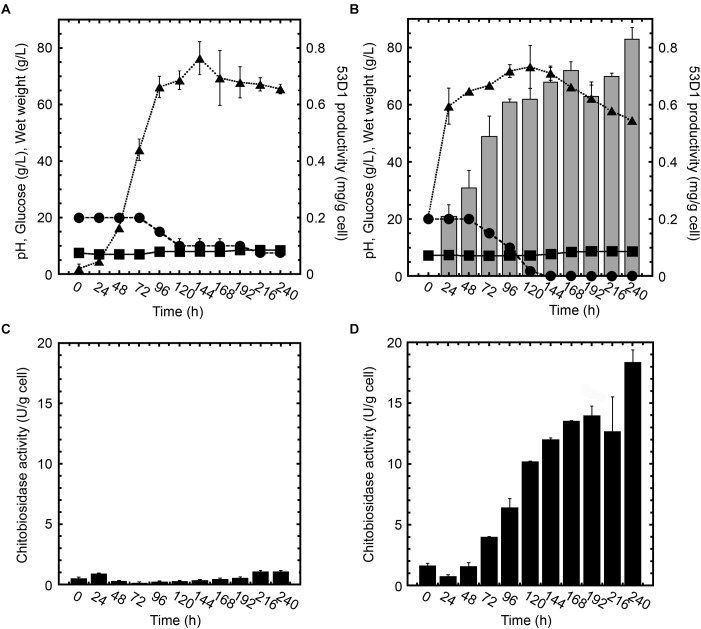
Growth curves, 53D1 production **(A,B)**, and chitinase activities **(C,D)** in *S. coelicolor*/pIJ86 (left panels) and *S. coelicolor*/pIJ86::*53D1* (right panels), grown in YEME medium. In panels **(A,B)**, wet weight (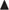, dotted line), pH (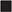, solid line), glucose consumption (•, dashed line), and 53D1 production determined by western blot analysis of cell-free culture broths (gray bars). In panels **(C,D)**, chitinase activity of cell-free culture broths measured by fluorimetric activity assay on 4-MU-(GlcNAc)_2_ as substrate (black bars).

*53D1* coding gene was thus cloned into the multicopy plasmid pIJ86 and introduced by intergeneric conjugation into *S. lividans* TK24, *S. venezuelae* ATCC 10595, and *S. coelicolor* A3(2). For selecting the best expression system, the three recombinant streptomycetes (and their control strains carrying empty vectors) were cultivated in five different media (see section “53D1 Heterologous Production”). Recombinant *S. lividans*/pIJ86::*53D1* did not produce the heterologous chitinase -neither inside nor outside the cells – in any of the cultivation media used (data not shown). 53D1 was instead secreted by the recombinant *S. venezuelae*/pIJ86::*53D1* growing in YEME medium (data not shown) and, to a major extent, by *S. coelicolor*/pIJ86::*53D1* cultivated in the same condition (Figure 2). Western blot analysis indicated that *S. venezuelae*/pIJ86::*53D1* produced a maximum of 8.75 mg/L of extracellular 53D1 (corresponding to 0.27 mg/g cell) (data not shown), whereas *S. coelicolor*/pIJ86::*53D1* secreted up to 45 mg/L (0.83 mg/g cell) of 53D1 (Figure 2B). No traces of 53D1 were detected into cytoplasmic soluble fractions of both the recombinant strains (data not shown).

Comparison of *S. coelicolor*/pIJ86::*53D1* (Figure 2B) growth curve with the one of its control strain carrying the empty vector (Figure 2A) indicated that *S. coelicolor*/pIJ86::*53D1* grew faster and consumed glucose more efficiently. This better performance of *S. coelicolor*/pIJ86::*53D1* was quite unexpected since the expression of heterologous genes usually causes a metabolic burden to the producing bacterial host, which slows down its growth rate (Binda et al., 2013). When observed at the optical microscope, the mycelium of *S. coelicolor*/pIJ86::*53D1* was less clumpy than in the control strain; this phenotype might be due to a putative lysozyme-like activity of 53D1. It has been demonstrated that lysozyme, producing a more disperse mycelium, facilitates streptomycetes growth in liquid media (Hobbs et al., 1989). A lysozyme activity of several chitinases was indeed previously reported by other authors (Bokma et al., 1997; Wohlkönig et al., 2010).

Cells of *S. coelicolor*/pIJ86::*53D1* started to secrete 53D1 after approximately the first 24 h of growth and continued to produce the heterologous protein during the stationary growth phase: the maximum specific productivity was reached after 240 h (Figure 2B). Consistently, in the same period of time, the chitinase enzyme activity measured in cell-free culture broths of *S. coelicolor*/pIJ86::*53D1* progressively increased and reached a maximum of ca. 18.5 U/g cell after 240 h (Figure 2D). As expected, no 53D1 was detectable by western blot analysis in the cell-free culture broths of *S. coelicolor*/pIJ86 (Figure 2A). The traces of chitinase activity detectable in the cell-free culture broths of the control strain (never exceeding the level 0.1 U/g cell; Figure 2C) were due to the endogenous streptomycetes chitinolytic system, opportunely repressed by the addition of glucose to the cultivation medium (Berini et al., 2018).

### 53D1 Purification and Characterization

**FIGURE 3:**
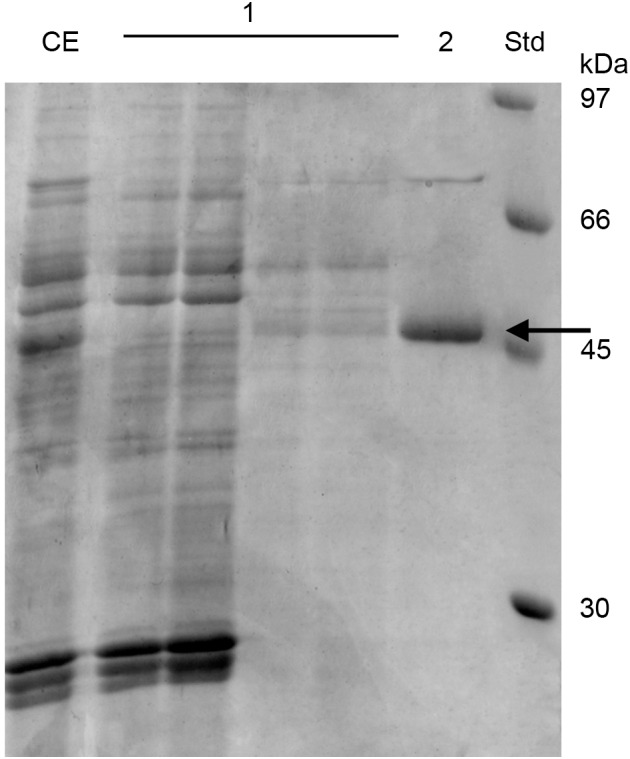
SDS-PAGE of 53D1 purification. CE, crude extract, i.e., *S. coelicolor*/pIJ86::*53D1* concentrated culture broth, loaded onto HiTrap-Chelating affinity column; 1, fractions collected from the initial washing of the column; 2, purified 53D1; Std, standard reference proteins. 53D1 protein band is indicated with an arrow.

53D1 was recovered from the culture broth of *S. coelicolor*/pIJ86::*53D1*, harvested after 192–240 h of growth in YEME medium, as described in the Sections “53D1 Heterologous Production” and “53D1 Purification.” His_6_-53D1 was then purified as a single band of ca. 44 kDa (44.40 kDa is the expected molecular mass for the recombinant His_6_-tagged protein) by means of HiTrap-chelating affinity chromatography, with a purity of ca. 90% (Figure 3). Purification yield was 34.9 mg/L (Table 1), corresponding to ca. 0.64 mg/g cell. Fluorimetric enzyme assay using standard synthetic oligosaccharides confirmed that 53D1 has a prevalent chitobiosidase activity on 4-MU-(GlcNAc)_2_ (31.60 ± 2.90 U/mg protein), a weaker endochitinase activity on 4-MU-(GlcNAc)_3_ (16.42 ± 1.85 U/mg protein), and none β-*N*-acetyl-glucosaminidase activity on 4-MU-GlcNAc. On colloidal chitin, the maximum activity of 53D1 was measured at pH 5.0, although the protein conserved ca. 70% of its maximum activity also at neutral and basic pH. It conserved ca. 27% of its initial activity at pH 3.0 (Table 2). None protease or lipase activity was detected in the enzyme preparation (data not shown).

### *In vitro* Effects of 53D1 and Chi18H8 on the PM of *B. mori* Larvae

**FIGURE 4:**
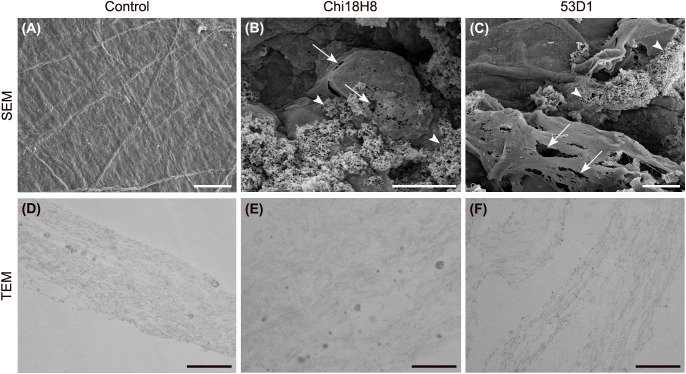
Morphology of the peritrophic matrix treated with chitinases. SEM **(A–C)** and TEM **(D–F)**. **(A,D)** Control samples; **(B,E)** in PM treated with Chi18H8, ruptures of the superficial layers (arrows) and alteration of the fibril network (arrowheads) are visible, as confirmed by TEM analysis; **(C,F)** similar effects can be observed in PM treated with 53D1. Bars: **(A–C)** 10 μm; **(D–F)** 0.5 μm.

To evaluate the potential insecticidal effects of 53D1 and Chi18H8, both chitinases were first tested *in vitro* by exposing the PM isolated from last instar larvae to a concentrated preparation of pure enzymes (40.5 U_tot_). SEM and TEM analyses of untreated PM (control) highlighted the well-organized and compact structure of *B. mori* PM: chitin fibrils were properly aligned and PM showed a continuous surface (Figure 4A,D). On the contrary, the analysis of the PM treated with Chi18H8 revealed a marked effect induced by the chitinase (Figure 4B,E). In particular, ruptures of the superficial layers (Figure 4B) and alteration of the integrity of the chitin network (Figure 4B,E) were clearly visible. The morphological analysis revealed a significant alteration of the structural organization of PM also when treated with 53D1 (Figure 4C,F). As for the PM treated with Chi18H8, the superficial layers of 53D1-treated PM were damaged (Figure 4C) and the disruption of the fibril network was visible (Figure 4F).

### *In vivo* Effects of 53D1 and Chi18H8 on *B. mori* Larvae

**FIGURE 5:**
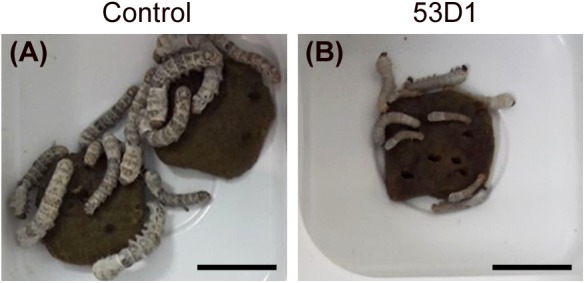
Pictures of *B. mori* larvae, on the 11th day after hatching, reared on artificial diet overlaid with 100 mM sodium acetate, pH 5 (**A**, control) or 6 U_tot_/cm^2^ of 53D1 dissolved in the same buffer **(B)**. All the control larvae are alive and the majority of them have reached the third instar **(A)**; a few larvae treated with the chitinase are already dead, and the majority of the survived larvae are still in the second instar **(B)**. Bars: 1 cm.

**FIGURE 6:**
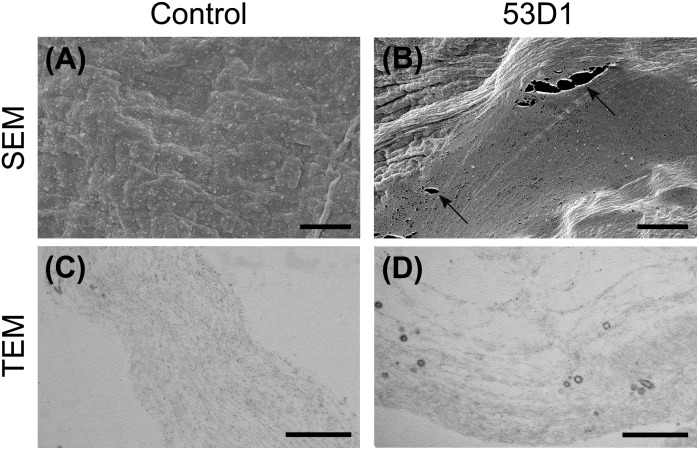
Morphology of the peritrophic matrix isolated from larvae treated with 53D1. SEM **(A,B)** and TEM **(C,D)**. **(A,C)** Control samples; **(B,D)** the treatment with 53D1 determines ruptures of the PM (arrows) and the disruption of the fibril network as confirmed by TEM analysis. Bars: **(A,B)** 5 μm; **(C,D)** 0.5 μm.

To evaluate the *in vivo* effects of 53D1 and Chi18H8, bioassays exposing the larvae of *B. mori* to chitinase-containing diet were conducted. The larval mortality, the length of the larval stage, and the weight of the pupae were not significantly different between untreated (control) and Chi18H8-treated larvae (Table 3). In contrast, the developmental parameters recorded for larvae fed with 53D1-containing diet indicated a clear detrimental effect of the chitinase (Table 4). In fact, the mortality of 53D1-treated larvae was significantly higher than in control larvae, the duration of the larval stage of the survived larvae was 25% longer, and their maximal larval weight before pupation was markedly reduced. As shown in Figure 5, the effect on larval development was visible from early instars onward. Moreover, pupal and cocoon weight was significantly lower in 53D1-treated larvae than in controls (Table 4). Finally, the PM isolated from survived last instar larvae reared on 53D1 chitinase-containing diet showed a compromised structure both at SEM and TEM (Figure 6). These effects on PM caused by 53D1 were comparable to those previously observed in *in vitro* experiments (see Figure 4), indicating that the alterations of the larval growth and development observed in the bioassay were due to the direct effect of 53D1 chitinase on PM.

**Table 3:** Effects of Chi18H8 on *B. mori* growth and development.

Doses of Chi18H8 (U_tot_/cm^2^ diet)	Larval mortality (%)	Duration of larval stage (days)	Pupal weight at day 8 (g)
0 (control)	0.00 ± 0.00	27.21 ± 0.27	1.32 ± 0.04
6	6.06 ± 3.03	27.58 ± 0.32	1.31 ± 0.05

*Each value represents the mean ± standard error of three independent experiments. Each experimental group was composed of 11 larvae. Larvae mortality is reported as the percentage of the initial number of larvae.*

**Table 4:** Effects of 53D1 on *B. mori* growth and development.

Doses of 53D1 (U_tot_/cm^2^ diet)	Larval mortality (%)	Duration of larval stage (days)	Maximal larval weight before pupation (g)	Pupal weight at day 8 (g)	Cocoon weight at day 8 (g)
0 (control)	2.78 ± 2.78	24.83 ± 0.21	3.32 ± 0.09	1.06 ± 0.04	0.24 ± 0.01
6	61.11 ± 2.78^∗^	31.69 ± 1.37^∗^	2.14 ± 0.15^∗^	0.80 ± 0.06^∗^	0.13 ± 0.01^∗^

*Each value represents the mean ± standard error of three independent experiments. Each experimental group was composed of 12 larvae. Larvae mortality is reported as the percentage of the initial number of larvae. ^∗^p < 0.001 versus control, Student’s t-test.*

### 53D1 and Chi18H8 Residual Activity in *B. mori* Midgut Juice

**FIGURE 7:**
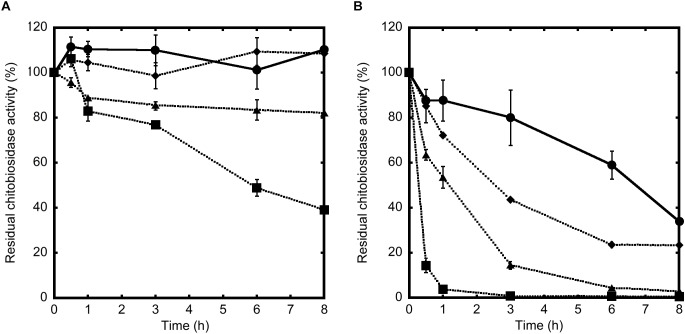
Residual chitinolytic activities of 53D1 **(A)** and Chi18H8 **(B)**, incubated at 25°C in Tris–HCl pH 8.0 (control, •, solid line) or in the presence of undiluted (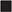, dotted line), 10-fold diluted (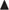, dotted line), or 100-fold diluted (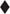, dotted line) midgut juice from *B. mori* larvae. Enzyme aliquots were collected at increasing time intervals and the residual activity was measured on 4-MU-(GlcNAc)_2_ as substrate.

To explain the different *in vivo* activity of the two chitinases, the residual enzyme activity of Chi18H8 and 53D1 was measured following their incubation for different time intervals in the absence or presence of midgut juice (at different dilutions) isolated from *B. mori* larvae. Indeed, the midgut juice from lepidopteran larvae has an alkaline pH and contains enzymes responsible for macromolecule digestion, including proteases (Terra and Ferreira, 1994). 53D1 activity was stable in the control buffer at alkaline pH 8 for at least 8 h (Figure 7A). In the presence of midgut juice, its residual activity was dependent on midgut juice dilution: anyhow, after 8 h of incubation with undiluted midgut juice the enzyme still retained ca. the 40% of its initial activity (Figure 7A). In contrast, the activity of Chi18H8 was much more drastically reduced by incubating the enzyme in the control buffer at alkaline pH and in the presence of midgut juice (Figure 7B). After 8 h in the control buffer, the residual activity was reduced to less than 40%. When incubated with 10- and 100-fold diluted midgut juice, the residual activity after 8 h was ca. 3 and 23% of the initial activity, respectively. In the presence of undiluted midgut juice, Chi18H8 completely lost its enzymatic activity within 1 h of incubation. These results indicated that the lack of *in vivo* effects of Chi18H8 in *B. mori* larvae was due to the loss of enzyme activity in the alkaline midgut juice environment, coupled with a probable proteolytic damage caused by the proteases present in the midgut lumen.

## Discussion

In the present work, we tested the insecticidal activity of two recently discovered soil metagenome-sourced chitinases on the larvae of *B. mori*, by using a combined *in vivo* and *in vitro* approach. *B. mori* is a model organism among Lepidoptera, which represent the second largest order of insects, including damaging phytophagous species that are still mainly controlled with chemicals. The two chitinases used in this study (Chi18H8 and 53D1) are diverse from all those described previously, possessing specific structural and functional features. Previous results both from sequence and substrate specificity analyses indicated that Chi18H8 belongs to family 18 of glycosyl hydrolases (GH18), showing less than 45% amino acid sequence identity to any known chitinase (Hjort et al., 2014). Additionally, Chi18H8 possesses an antifungal activity which is uncommon among GH18 chitinases (Hjort et al., 2014; Berini et al., 2017b). This protein seems enough stable to be used in semi-field or field applications, since its range of activity appears adequate for inhibiting fungal phytopathogens growing in acidic and mesophilic environments (Hjort et al., 2014; Berini et al., 2017b). Also 53D1 belongs to GH18 chitinases, showing less than 46% amino acid sequence identity to any known chitinase. It probably derives from an uncultivable bacterium related to the *Chloroflexus* species *Nitrolancetus hollandicus* and *Ktedonobacter racemifer* (Cretoiu et al., 2015). Although a more complete characterization of 53D1 was hampered by the poor production yield of its recombinant form in *E. coli* (see below), previous studies showed that this protein tolerates elevated levels of NaCl: since its activity increases at higher salt levels, 53D1 is considered an uncommon halophilic (rather than halotolerant) chitinase (Cretoiu et al., 2015).

Initially, the major bottleneck to testing insecticidal activity of the two metagenome-sourced chitinases was providing the milligrams needed to perform *in vitro* and *in vivo* assay in *B. mori*. Unfortunately, there is not a highly predictable, all-purpose, and rational protocol to succeed in metagenome-sourced protein expression. Each protein requires the development of its own tailored production process and the selection of the more adequate expression host (Davy et al., 2017). *E. coli* still remains the first-choice host for protein production, but intrinsic limits of this bacterium are its poor secretory machinery and its tendency to accumulate heterologous proteins into IBs, mostly in inactive form. In the case of Chi18H8, we could recover hundreds of milligrams of pure and active chitinase from processing IBs, following a previously developed and scaled-up process (Berini et al., 2017b), but this approach was not transferable to 53D1 production. In fact, it is widely recognized that the outcome of IB processing is unpredictable and has to be empirically determined for each protein (de Marco et al., 2019; Slouka et al., 2019). After some unsuccessful attempts, 53D1 was finally successfully expressed in *S. coelicolor* A3(2), although its codon usage was slightly different from the one of streptomycetes [63% G+C content for *53D1* gene vs. ca. 72% for *S. coelicolor* A3(2) genome] (Kieser et al., 2000). The production level in *S. coelicolor* A3(2) was satisfactory (around 45 mg/L) and the heterologous protein was entirely secreted into the culture broth, thus markedly facilitating its recovery and purification. A single step of affinity chromatography allowed us to recover ca. 35 mg/L of highly pure protein, with a 60-fold improvement in volumetric yield when compared to *E. coli*. Streptomycetes, although still relatively poorly explored for the expression of heterologous chitinases, have important advantages versus *E. coli*. They are non-pathogenic microorganisms, commonly inhabiting soil, where they establish beneficial interactions with plants, by modulating plant defense mechanisms or facilitating symbioses between plant roots and beneficial microbes (Schrey and Tarkka, 2008). Additionally, streptomycetes are already commonly used as components of commercial soil amendments for bioremediation (Sharma et al., 2016; Cuozzo et al., 2018) or biocontrol (González-García et al., 2019; Olanrewaju and Babalola, 2019) and they are generally considered safe for agricultural use. Using this environment-friendly expression system for producing chitinases might represent a further advantage to support their sustainable development as promising insecticide proteins.

Once the supply issue of both proteins was overcome, we decided to test the insecticidal activity of the two pure preparations of Chi18H8 or 53D1 using the PM of *B. mori* as *in vitro* and *in vivo* target. Insects offer two potential targets for chitinases: cuticle, which consists of a pluristratified structure mainly formed by proteins and chitin chains, and PM, where chitin fibrils act as a scaffold for binding glycoproteins and proteoglycans. Both structures exert fundamental roles for the insect survival. Cuticle protects insects from parasites, pathogens, and dangerous chemicals, while allowing muscle attachment and preventing water loss from the body (Moussian, 2010). PM helps in the compartmentalization of digestive processes, protecting the midgut epithelium against abrasive food particles and defending the insect from ingested pathogens (Hegedus et al., 2009). Previous works recently reviewed in Berini et al. (2018) reported that entomopathogenicity of microbial strains is mediated by a cocktail of cuticle-hydrolyzing enzymes, which include chitinases. Indeed, the topical insecticide potential of these enzyme combinations is often limited due to the long time required for their action, the need of high local concentrations, and their poor stability and persistence in changing environmental conditions. A more promising perspective seems to be using chitinases for targeting PM via oral ingestion (Berini et al., 2016, 2018). An advantage of this approach is that chitinases might be formulated with other insecticidal molecules, facilitating their adsorption/penetration into the midgut epithelium and thus increasing their activity. For instance, the combined oral administration of chitinases with *Bacillus thuringiensis* δ-endotoxin crystal proteins was reported to dramatically enhance the toxic effect of the latter (Regev et al., 1996; Liu et al., 2010). Additionally, the insecticide activity of TMOF, a peptide that inhibits trypsin synthesis, was increased by combined administration with a viral chitinase (Fiandra et al., 2010).

Our results demonstrated that when the PM of the silkworm was exposed *in vitro* to chitinases, the combination of endo- and exo-activities possessed by both enzymes significantly altered the structure of PM, disrupting the organization of chitin fibrils. Peeling of the superficial layers, ruptures, separation of the fibril networks, and a general weakening of the PM were observed. The effects of the two enzymes were similar, although 53D1 appeared to cause a more marked damage to PM structure. This result was consistent with the demonstrated 53D1 greater activity on colloidal chitin, which mimics the complex insoluble-chitin-containing natural structures. Once orally administered to *B. mori* larvae, 53D1 induced mortality, enhanced dramatically the duration of the larval stage, and reduced both the maximal larval weight before pupation and pupal and cocoon weight, whereas Chi18H8 did not provoke any consequences on insect development. Ultrastructural analysis of PMs isolated from larvae reared on 53D1-containing diet, showed significant alterations, confirming that the structural damage of this matrix dramatically affected insect development probably due to a reduced nutrient digestion capability. The different *in vivo* activity between Chi18H8 and 53D1, which might appear puzzling considering that both the enzymes disrupted (although at a different extent) the PM integrity *in vitro*, became understandable once the poor residual activity of Chi18H8 in the alkaline and proteolytic environment of Lepidoptera midgut lumen was demonstrated. Apparently, the intrinsic properties of 53D1 made this enzyme less susceptible to degradation in the above-mentioned conditions. Although the administration of both chitinases to other insects, especially to those having a midgut lumen with neutral or acidic pH, is worthy to be investigated, this work demonstrates that actually 53D1 can be considered a more promising candidate than Chi18H8 as insecticide protein for oral administration. Fortunately, 53D1 further *in vivo* and in-field trials will be possible due to the development of a reliable and sustainable production process using as expression platform the unconventional -but more environmentally acceptable-*S. coelicolor*.

In conclusion, this work shed light on (i) the efficacy of metagenomic investigations for discovering novel enzymes to be implemented as part of integrated pest management programs; (ii) the potential of metagenome-sourced microbial chitinases as promising insecticide proteins; and (iii) the need to develop unconventional heterologous expression platforms to support insecticide protein development and use. Although insecticide formulations based on chemically synthesized compounds still represent a relevant part of crop protection, it is undeniable that insecticide proteins will contribute in future to the progressive reduction of chemicals, introducing novel strategies for managing insect pests. Formulation of chitinases with other biopesticides or chemically synthesized pesticides might allow the reduction of the environmental impact of single toxic compounds and reduce the risk of resistance selection (Chandler et al., 2011; Hardy, 2014). Microbial biotechnology will be crucial to support the development and sustainable production of novel insecticide proteins.

## Data Availability

All datasets generated for this study are included in the manuscript and/or the supplementary files.

## Author Contributions

FB, MC, GT, and FM conceived the experiments, interpreted the results, and wrote the manuscript. FB cloned the metagenome-sourced genes and produced the metagenome-sourced chitinases. AM and GT tested the insecticide proteins *in vitro*. MC tested the insecticide proteins *in vivo*. MR performed the microscopical observations. FM and GT managed the project. All authors reviewed and approved the final manuscript.

## Conflict of Interest Statement

The authors declare that the research was conducted in the absence of any commercial or financial relationships that could be construed as a potential conflict of interest.
